# Factors influencing consumer adoption of USB-based Personal Health Records in Taiwan

**DOI:** 10.1186/1472-6963-12-277

**Published:** 2012-08-27

**Authors:** Wen-Shan Jian, Shabbir Syed-Abdul, Sanjay P Sood, Peisan Lee, Min-Huei Hsu, Cheng-Hsun Ho, Yu-Chuan Li, Hsyien-Chia Wen

**Affiliations:** 1 School of Health Care Administration, Taipei Medical University, Taipei, Taiwan; 2 Institute of Biomedical Informatics, National Yang-Ming University, Taipei, Taiwan; 3 Health Informatics & Electronics Division, Centre for Development of Advanced Computing, Mohali, India; 4 Graduate Institute of Information Management, National Taipei University, New Taipei, Taiwan; 5 College of Medical Science and Technology, Taipei Medical University, Taipei, Taiwan

**Keywords:** Personal Health Records (PHR), Technology Acceptance Model (TAM), Adoption, Behavior, Taiwan

## Abstract

**Background:**

Usually patients receive healthcare services from multiple hospitals, and consequently their healthcare data are dispersed over many facilities’ paper and electronic-based record systems. Therefore, many countries have encouraged the research on data interoperability, access, and patient authorization. This study is an important part of a national project to build an information exchange environment for cross-hospital digital medical records carried out by the Department of Health (DOH) of Taiwan in May 2008. The key objective of the core project is to set up a portable data exchange environment in order to enable people to maintain and own their essential health information.

This study is aimed at exploring the factors influencing behavior and adoption of USB-based Personal Health Records (PHR) in Taiwan.

**Methods:**

Quota sampling was used, and structured questionnaires were distributed to the outpatient department at ten medical centers which participated in the DOH project to establish the information exchange environment across hospitals. A total of 3000 questionnaires were distributed and 1549 responses were collected, out of those 1465 were valid, accumulating the response rate to 48.83%.

**Results:**

1025 out of 1465 respondents had expressed their willingness to apply for the USB-PHR. Detailed analysis of the data reflected that there was a remarkable difference in the “usage intention” between the PHR adopters and non-adopters (χ2 =182.4, p < 0.001). From the result of multivariate logistic regression analyses, we found the key factors affecting patients’ adoption pattern were Usage Intention (OR, 9.43, 95%C.I., 5.87-15.16), Perceived Usefulness (OR, 1.60; 95%C.I., 1.11-2.29) and Subjective Norm (OR, 1.47; 95%C.I., 1.21-1.78).

**Conclusions:**

Higher Usage Intentions, Perceived Usefulness and Subjective Norm of patients were found to be the key factors influencing PHR adoption. Thus, we suggest that government and hospitals should promote the potential usefulness of PHR, and physicians should encourage patients' to adopt the PHR.

## Background

Usually patients receive healthcare services from multiple hospitals, and consequently their healthcare data are dispersed over many facilities’ paper and electronic-based record systems [1]. This fragmented system of storing and retrieving essential patient data impedes continuum care [2]. In the UK, Australia and New Zealand, the strategic goal has been to enhance electronic communication links between the primary care sector and secondary care institutions [3]. Research in Denmark showed patients wanting to seek more control over their personal health information [4]. Similarly, many countries are focusing on data interoperability, access, and patient's authorization [5]. The American Academy of Pediatrics supports development of educational programs for families and clinicians to promote effective and efficient use of the personal version of Electronic Health Records, called Personal Health Records (PHRs) as a way of improving the quality of healthcare for children [6]. For such reasons, Personal Health Records are gradually gaining grounds to the extent that companies like Microsoft have ventured into the world of Personal Health Records and it may be apt to expect PHRs getting integrated into clinical practice gradually.

Drawing on models of health IT development for advanced countries, Taiwan’s Department of Health (DOH) has initiated a five year project called the National Healthcare Information Project (NHIP) to promote adoption of the PHR system and to enhance health information exchange [7]. The DOH of Taiwan has created the Taiwan Electronic Medical Record Template (TMT) primarily to achieve functional and semantic interoperability of health information within the country. TMT is a local electronic record template that has been developed by adopting international standards such as HL7 Clinical Document Architecture (CDA), which is supposed to provide interoperability within healthcare systems [8]. The present research is centered on a USB-based Health Records project that promises to develop a fully interoperable and portable PHR system in Taiwan [9].

Taiwan’s Health Insurance system (implemented in 1995) is globally appreciated and renowned for its universal coverage. The government made enrollment compulsory for all citizens and legal residents. The Bureau of National Health Insurance (BNHI) is the single payer for about 19,000 healthcare providers in Taiwan. Patients have freedom to visit any type of healthcare provider, meaning referral from health centers and General Practitioners clinics is not required to visit medical centers. The tools like PHR could be helpful in providing timely medical history of the patient, thus eliminating unnecessary tests requests and drug prescriptions when patients visit different healthcare providers. Taiwan thus provides a favorable environment to take maximum advantages of the PHRs.

Although the term “Electronic Health Record” is widely used, there are other terms such as Personal Health Record and Electronic Medical Record associated with it [10]; however, in academics it is inappropriate to use these terms interchangeability. In this study our definition of PHR is limited only to the information related to past, present and future medical condition of the patient — the information essential for providing health care. Personal Health Records are expected to engage consumers into their own care management by empowering them with tools and knowledge that would facilitate their access and interaction with different hospitals in a more efficient and effective manner. USB-PHR is a Universal Serial Bus flash drive that provides encrypted flash memory for secure storage and access to health records.

Studies have explored electronic record technologies and functionalities that are focused on opinions of medical personnel, such as physicians and nurses [11]. Whiddett et al. [3] pointed out that patients’ attitudes towards new technology and willingness to share their health information are important factors for the successful adoption of electronic records. Extant published literature posits that adoption of EHRs and PHRs continues to be slow, as researchers and practitioners have cited numerous factors those influence patient’s adoption of PHRs [12]. However, this study is aimed to investigate the factors affecting patient adoption behavior towards PHR. Results of this study will be beneficial in understanding user perspectives and supporting widespread adoption of PHRs by individuals.

### Research model

The surge in health IT implementation projects has elevated interest among the researchers towards the theories that explain intention and adoption behaviors of the end users towards technologies in the healthcare sector. Technology Acceptance Model (TAM) is considered to be one of the well-studied and tested theories of technology acceptance [13]. We have referred to TAM scale of Davis [14], Social norm questionnaire of Venkatesh [15] and self-computer effectiveness scale from Murphy, Coover and Owen [16] and included the notion of security and privacy in the questionnaire. The rationale for using TAM is to evaluate technology (in this study PHR) acceptance and Theory of Planned Behavior (TPB) that links the interaction between intention and behavior of the samples in the study. Our research tries to enrich the existing technology acceptance literature by adding security and privacy characteristics, thus providing a platform for investigating more comprehensive system characteristics influencing PHR adoption.

**Figure 1:**
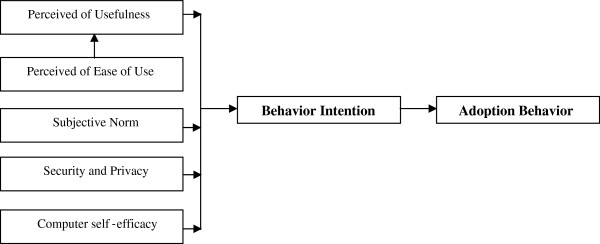
Research model.

Based on the TAM, TPB and the reviewed literature, our proposed research model is depicted in the Figure1. According to this model, Adoption Behavior (AB) and Behavior Intention (BI) are a function of five concrete variables: Perceived Usefulness (PU), Perceived Ease of Use (PEOU), Subjective Norm (SN), Computer Self-Efficacy (CE) and Security and Privacy (SP).

Perceived usefulness is defined as “the degree to which a person believes that using a particular system would enhance his or her job performance.” Perceived ease of use refers to “the degree to which a person believes that using a particular system would be free of effort” [14]. Computer self-efficacy is associated with beliefs and behaviour towards computer usage and it impacts the usage of computer technology [17]. Subjective norm is defined as “a person’s perception that most people who are important to him think he should or should not perform the behavior in question” [18].

Since electronic health records is an emerging application of health information technology in Taiwan, our study is pivoted on the suggestion of Venkatesh et al. [15] that the research model for acceptance of information technology could be based jointly on TAM and TPB. This will enhance the understanding about the user intent and adoption behaviour. Thus, the questionnaire is based on the TAM and TPB with new characteristics pertaining to the individual’s concern for information privacy and security being included in our study. In order for USB-PHR to become generally acceptable to consumers, privacy and security can directly influence the behavioural intentions hence, these issues must be addressed [19].

## Method

### Participant enrollment

**Figure 2:**
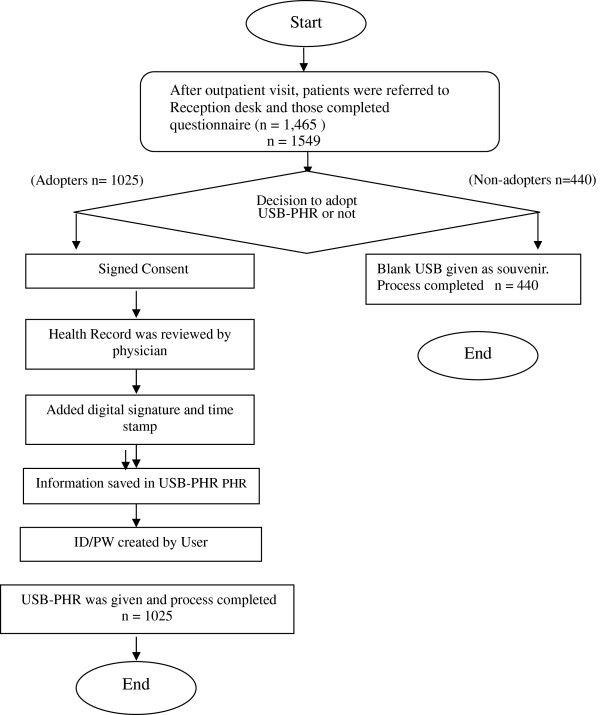
The study design.

This study includes 10 medical centers (teaching hospitals) in Taiwan that had volunteered to participate in the Taiwan DOH TMT project. The research assistant distributed 300 questionnaires at the reception desk of each of these participating medical centers. After outpatient consultation, patients were asked about their willingness to participate in the study, and had to spare some time to learn about the USB-PHR system as well as to respond to the questionnaire. The patients that responded to the questionnaire were included in the study after signing a consent form. The patients who were interested in applying for a USB-PHR were adopters and those who didn’t apply were non-adopters. The adopters had to sign an additional consent form stating that they were responsible for the information stored in the USB-PHR. It took about two months to reach the desired subject number of 1,500 samples. Altogether 1,549 questionnaires were collected, and out of these 1,465 were valid respondents; therefore the effective response rate was 48.83%. Figure2 shows the design of the study. A written consent was taken from each participant after explaining the purpose of this study. This study was approved by the IRB of Taipei Medical University.

### Measures and data analysis

The questionnaire was based on the Technology Acceptance Model (TAM) and Theory of Planned Behavior (TPB), and it passed both validity and reliability testing from the studies evaluating adoption of on-line tax and Internet banking [20,21]. After adjusting for patient characteristics, multivariate logistic regression analyses were also employed to assess the association between independent variables and PHR adoption behavior. The 5-point Likert scale was used to score the questions in which the respondents were asked to agree or disagree based on a ranking from 5 to 1, where 5 was strongly agree and 1 for strongly disagree. However, for User Intention, previous experience with paper-based medical record and hospitalization or referral was asked in ‘Yes’ or ‘No’ as binary variables. Questions were chosen as per the following criteria: content validity index above 0.8 and Cronbach’s α of four fifths of constructs were above 0.83, except when “privacy and safety” was 0.708. SPSS version 12.0 was used to perform statistical analysis of the data. Variables like demographic information, Perceived Usefulness, Ease of Use, Subjective Norm, Privacy & Security, User Intension and Computer Self-efficacy were collected through questionnaires. Chi-square analysis and independent t test were used for comparisons between PHR adopters and non-adopters. Correlation analysis was adopted to understand the correlations between variables. The score of concept’s questions represented each concept in the multiple regression statistics. The questionnaires and the description of USB-PHR system were distributed in Mandarin. However, English translation was presented in the Additional file 1: appendix.

### USB-PHR system

**Figure 3:**
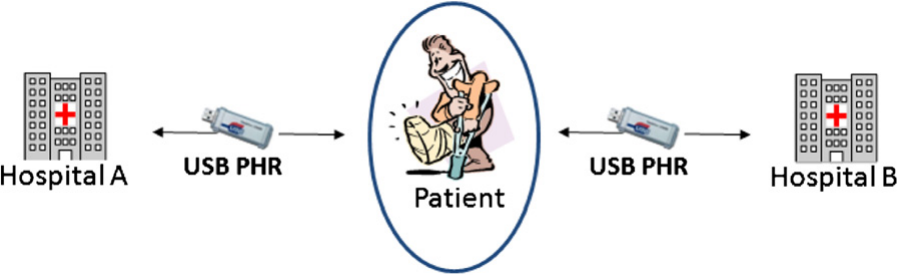
Showing information collection from different hospitals.

The USB-PHR system is a portable personal health summary that stores a minimal data set of medical information essential for providing health care. The USB flash drive given to the patients contains a software to collect and view their personal medical information such as medication, lab results, multimedia files like endoscopy or ultrasound scanning recordings from hospitals from which they received treatment. Information will be updated from the reception desk when the patients are discharged or after outpatient visit from the participating hospitals. The overall design architecture is presented in our other paper [9]. In this paper a general concept of the design is presented. USB-PHR contains two components, namely, XML data component and the viewer. The data component is a set of XML files compliant to a standard called TMT (Taiwan electronic Medical records Template) that is a derivative of HL7 CDA-R2. This XML data component helps in collecting patients’ relevant information from hospitals’ information system. Using the USB-PHR patients can view the encrypted information that is provided by participating hospitals. Figure3 shows the information collection from different hospitals.

**Figure 4:**
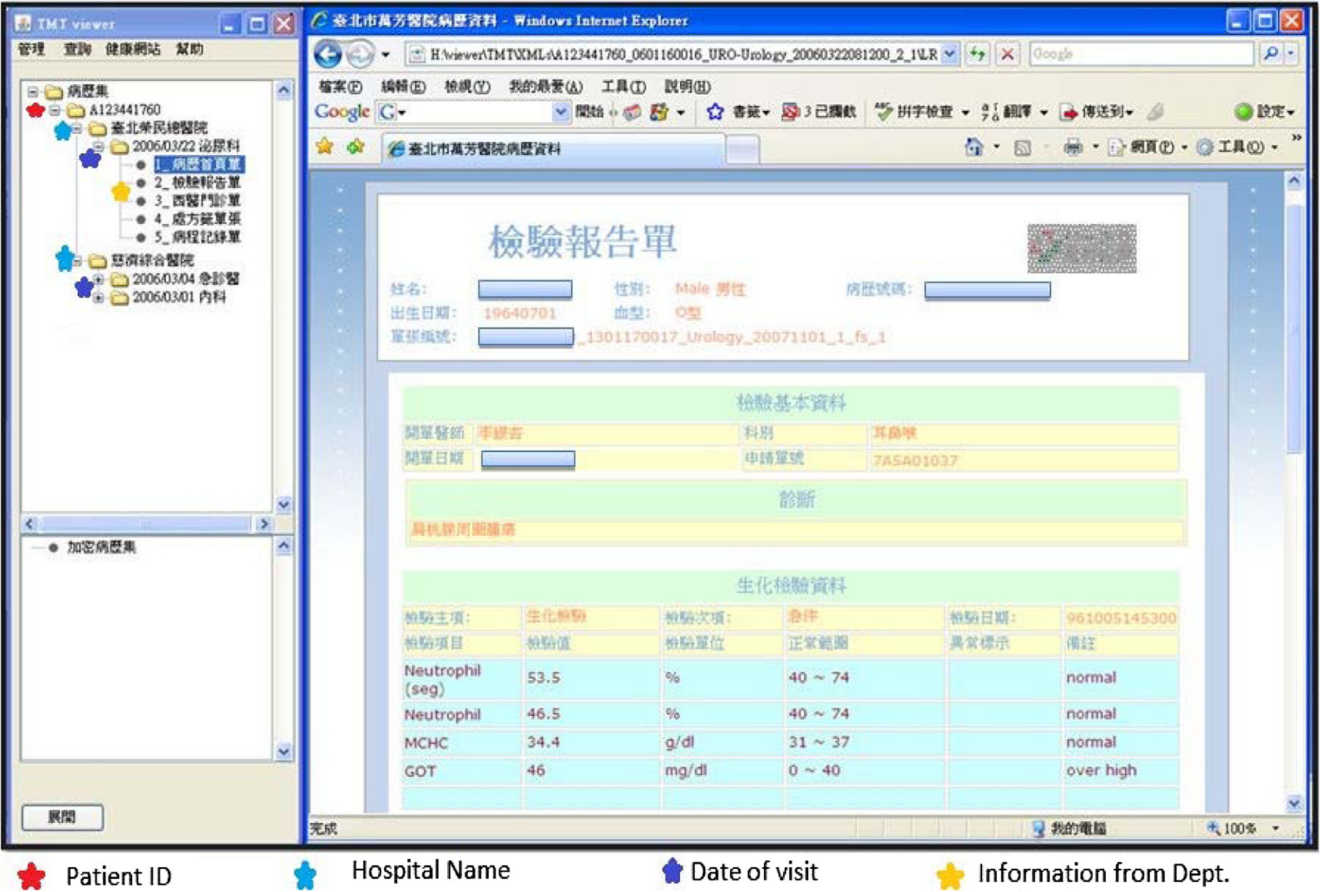
User Interface of PHR displaying lab results.

This system is made to handle information from multiple hospitals. Figure4 is screenshot of the viewer showing personal lab results when the patient plugs-in the USB into a PC or laptop and opens the viewer by entering password.

## Results

Table1 shows the distribution of the sampled patients’ characteristics. Of the respondents, 941 (64.2%) were female and 524 (35.8%) were male with the highest percentage of the patients aged between 30 and 39 years (31.0%) and most had university level education (38.8%).

**Table 1:** Demographic characteristics of responding patients (n = 1,465)

**Variables**	**N**	**(%)**
**Gender**		
Male	524	35.8
Female	941	64.2
**Age**		
Under 29 years old	374	25.8
30-39 years old	449	31.0
40-49 years old	345	23.8
50-59 years old	172	11.8
Above 60 years old	110	7.6
**Education levels**		
Under junior high	161	11.1
High school	182	12.5
College	351	24.2
University	563	38.8
Graduate school	195	13.4

Bivariate analyses were used to examine whether 1,025 PHR adopters differed significantly from 440 non-adopters (Table2). The chi-square tests were conducted by usage intention and experiences of medical treatment. The results show statistically significant relationships in “usage intention” (*χ*^*2*^ = 182.4, *p* < 0.001), “experience of releasing paper-based medical record” (*χ*^*2*^ = 11.54, *p* < 0.001) and “experience of hospitalization or referral” (*χ*^*2*^ = 6.95, *p* < 0.01).

**Table 2:** Usage intention and experiences of hospitalization influencing patients’ adoption of PHR

	**Adopters**		**Non-adopters**		
**Variances**	**n = 1025**	**%**	**n =440**	**%**	χ^**2**^
Usage intention					182.4^c^
Yes	994	97.0	323	73.9	
No	31	3.0	114	26.1	
Experienced release of paper-based medical record					11.54^c^
Yes	389	38.3	127	29.0	
No	627	61.7	311	71.0	
Experiences of hospitalization or referral					6.95^b^
Yes	521	51.0	191	43.5	
No	500	49.0	248	56.5	

^a^ p < 0.05 ; ^b^ p < 0.01;^c^ p < 0.001.

In multivariate logistic regression (Table3), the adjusted odds ratio (OR) showed patients who were above 40 years of age were more prone to adopt PHR, while for age above 60 years the OR was 3.06 95%C.l. (confidence interval 1.27-7.33) compared with the under 29 year old age group. However, participants with higher educational levels have shown less interest towards PHR adoption (OR, 0.30 C.l. 0.18-0.81). Participants reporting higher usage intention were more likely to adopt PHR (OR 9.43, 95%C.I. 5.87-15.16). Higher perceived usefulness (OR, 1.60; 95%C.I., 1.11-2.29) and subjective norms (OR, 1.47; 95%C.I., 1.21-1.78) increased adoption of PHR. However, when computer self-efficacy was compared, PHR adopters were significantly lower than non-adopters (OR, 0.77, 95%C.I., 0.59-0.99).

**Table 3:** Multivariate logistic regression of predictors of PHR adoption

**Independent variables**	**Odds ratio**	**95% C.I.**
**gender** (female)		
Male	1.19^d^	0.89-1.61
**age** (under 29 years old)		
30-39 years old	0.94^d^	0.66-1.34
40-49 years old	1.52^a^	1.01-2.27
50-59 years old	1.61^a^	1.06-2.72
above 60 years old	3.06^a^	1.27-7.33
**Education level** (under junior high school)		
High school	0.30^b^	0.14-0.64
College	0.34^b^	0.17-0.69
University	0.36^b^	0.18-0.73
Graduate school	0.38^a^	0.18-0.81
**Usage intention** (no)		
Yes	9.43^c^	5.87-15.16
**Perceived usefulness**	1.60^a^	1.11-2.29
**Perceived ease of use**	1.29^d^	0.95-1.75
**Subjective norm**	1.47^c^	1.21-1.78
**Privacy and safety**	0.83^d^	0.61-1.13
**Computer self-efficacy**	0.77^a^	0.59-0.99

The item in parentheses is reference.

^a^ p < 0.05 ; ^b^ p < 0.01;^c^ p <0.001; ^d^ p > 0.05.

## Discussion

The key objective of the National Healthcare Information project is to set up a portable data exchange environment in order to enable people to maintain and own their health records. Therefore, this study is aimed at exploring the factors influencing adoption of Personal Health Records (PHR) by its users. This study reveals that higher Usage Intentions, Perceived Usefulness and Subjective Norm of patients were found to be the key factors influencing PHR adoption.

The Usage intention is an important factor that seems to influence their adoption behavior. Table2 illustrates that 97% of patients had intention to use the new patient records technology, and the chi-square test showed significance in this data (χ^2^ = 182.4, p < 0.001). From the multivariate logistic regression (Table3), we also found that a patient’s intention to use the system was the most effective factor effecting adoption behavior (OR = 9.431, P < 0.001). This is similar to the TAM model’s results showing that “usage intention” is the primary factor in acceptance [16-18]. Therefore, we believe that arousing patients’ intention to use electronic health records would be an effective way to increase adoption rate. Furthermore, Table2 also showed that a total of 1,317 (89.9%) patients had intention to use USB-PHR, but only 1,025 (69.96%) patients adopted it, which revealed that 20% of patients did not take action. A similar result was reported by the Personal Health Working Group (2003), which found that patients have high usage intentions regarding PHR, but that the adoption rate is relatively lower among patients who have been offered access to an PHR, even though it has been offered free to them [22].

In a TAM model perceived usefulness was a significant factor for technology acceptance [14]. We found that perceived usefulness affected adoption behavior positively, and this finding concurs with other studies where perceived usefulness influenced or correlated with usage intention and actual user behavior [23-25]. Under the design of TMT infrastructure, the functionalities of our USB-PHR were to provide interoperability within healthcare providers, to store summaries of health information, to maintain patients’ current medications, and to keep track of known adverse reactions and known allergies. We also believe that the usefulness of the PHRs could be enhanced further with online access and on the mobile phone.

Tang et al. [1] suggested that the most-anticipated Internet applications include access to information on new treatments, e-mail communication, and medication information. Furthermore, Maloney and Wright identified and analyzed thirteen USB based PHRs in US and found PHRs currently available in the market appear to have deficiencies; therefore, they suggested tethered or web-based PHRs like Microsoft HealthVault platform might be a better option for consumers at present [26]. However, we believe that USB-PHR could be adopted as one of the useful options specifically in a poor Internet connectivity context if its functionality is well designed and meets patients' concerns.

In the TPB model, subjective norms can change usage intention positively [27]. Our study likewise shows that patients’ adoption behavior will be influenced positively by subjective norms (OR = 1.47, p = 0.001). Finally, we must be aware that policy changes will likely lead to improved consumer adoption of PHR, such as expanding its functionality, establishing standards for PHR information, facilitating the unencumbered secure exchange of health information, improving consumers’ access to PHR, and helping consumers improve their understanding of the information contained in a PHR [28].

The acceptance of PHR was also influenced by age, education and computer self-efficacy. It is interesting to note that the older patients whose education levels were under junior high school and with lower computer self-efficacy were more likely to request USB-PHR. We thought the possible reasons for such result could be that USB-PHR was convenient for older patients to carry. They need not worry anymore about remembering their medical history and medication schedules, but rather have access to their medical information anytime they need. However, Venkatesh et al. [15] found that older workers were less willing to adopt new IT products. Age could also affect usage intention directly or affect indirectly through ease of use [29]. Education was an effective demographic characteristic that improved the ability to understand information and learn from the experiences [30,31] that could indirectly affect willingness through perceived ease of use. Computer self-efficacy certainly affects usage of new IT [32,33] and was an intervening variable between environmental factors and usage behavior [17].

We found that “Education Level”, “Computer self-efficacy” and “Privacy and Safety” each have an odds ratio significantly below one (see Table3). This indicates that people with higher education and better computer self-efficacy might be more concerned about privacy; therefore they were reluctant towards adopting PHR. We may need strong evidence with better explanation of its usefulness and security technologies used in order to convince them to adopt PHRs. Our assumption of the odds ratio to be below one for all the education levels isthat the majority of the elderly population has an education level under junior high school. In addition, one more point to be considered is that the younger age group (below 39 years) respondents might not be requiring healthcare services as much as the elderly age group (above 60 years) may require. Furthermore, the nature of the ailments of the younger population may be trivial, thereby reflecting low perceived usefulness of USB-PHR for them.

## Conclusion

This study showed that strong “Usage Intention” among patients was the key factor in adopting USB-PHR. “Subjective norms” and “Perceived usefulness” were also critical issues for the adoption of PHR. Therefore, we suggest that government and hospitals continuously promote educational campaigns devoted to clinical informatics in medical schools, on job education to help both patients and clinicians to understand the potential advantages and limitations of the USB-PHR. Researchers should not only focus on improving the functionalities but also on addressing the patient concerns about privacy and security while seeking referrals or medical care in multiple hospitals. As a bottom line we suggest that government and hospitals should propagate users about the potential benefits of PHR, and physicians should encourage patients to adopt PHR. This approach would effectively increase patients’ adoption of the PHR.

### Study limitations

Due to time and resource limits, this study only sampled outpatients from 10 medical centers in Taiwan. In addition, we feel that the research model may also be tested for reliability and validity with different user groups in different settings with various information seeking contexts. However, this study gave us the platform for further evaluating users’ intention and adoption behavior towards PHR.

## Misc

Wen-Shan Jian, Shabbir Syed-Abdul, Yu-Chuan Li and Hsyien-Chia Wen these authors contributed equally to this work

## Competing interests

The authors declare that they have no competing interests.

## Authors’ contribution

W-SJ participated in the study design, performed and helped to draft the manuscript. SS-A carried out analysis and interpretation of the data and drafted the manuscript. SPS helped in drafting of the manuscript and statistical analysis. PL helped in execution of the study, collection of the data, analysis and interpretation. M-HH participated in the study design and conceived of the study. C-HH involved in drafting of the manuscript and statistical analysis. Y-CL participated in the study design, performed and helped in interpretation of the data, supervised the drafting of the manuscript. H-CW participated in the study design and conceived of the study, involved in statistical analysis and interpretation. All authors read and approved the final manuscript.

## Pre-publication history

The pre-publication history for this paper can be accessed here:

http://www.biomedcentral.com/1472-6963/12/277/prepub

## Supplementary Material

Additional file 1 Appendix. Click here for file

## References

[B1] McDonald CJ Overhage JM Dexter PR Canopy computing: using the Web in clinical practice JAMA 1998 280 1325 1329 10.1001/jama.280.15.1325 9794311

[B2] Tang PC Ash JS Bates DW Overhage JM Sands DZ Personal health records: definitions, benefits, and strategies for overcoming barriers to adoption J Am Med Inform Assoc 2006 13 121 126 10.1197/jamia.M2025 16357345 PMC1447551

[B3] Whiddett R Hunter I Handy JE Patients' attitudes towards sharing their health information Int J Med Inform 2006 75 7 530 541 10.1016/j.ijmedinf.2005.08.009 16198142

[B4] Zurita L Nohr C Patient opinion–EHR assessment from the users perspective Stud Health Technol Inform 2004 107 Pt 2 1333 1336 15361031

[B5] Sood SP Nwabueze SN Mbarika VWA Prakash N Chatterjee S Ray P Electronic medical record: A review comparing the challenges in developed and developing countries 2008 Washington: IEEE Computer Society 248

[B6] Schneider JH Marcus E Del Beccaro MA Using Personal Health Records to Improve the Quality of Health Care for Children Pediatrics 2009 124 1 403 409 19564327 10.1542/peds.2009-1005

[B7] Chang I Stakeholder Perspectives on Electronic Health Record Adoption in Taiwan Manage Rev 2010 15 1 133 145

[B8] Jian WS Hsu CY Hao TH Wen HC Hsu MH Lee YL Li YC Chang P Building a portable data and information interoperability infrastructure-framework for a standard Taiwan Electronic Medical Record Template Comput Methods Programs Biomed 2007 88 102 111 10.1016/j.cmpb.2007.07.014 17936402

[B9] Jian WS Wen HC Scholl J Shabbir SA Lee P Hsu C-Y Li Y-C The Taiwanese method for providing patients data from multiple hospital EHR systems J Biomed Inform 2011 44 2 326 332 10.1016/j.jbi.2010.11.004 21118726

[B10] Murphy GF Electronic Health Records: Changing the Vision 1999 Philadelphia: Harcourt Brace & Co

[B11] Sara K Jeremy G David M Evidence Summary: Electronic Health Records (EHRs) 2010 Ottawa: Champlain LHIN http://www.docstoc.com/docs/53410184/KTA--EHR--Evidence--Review

[B12] Trisha G Susan H Katja S Tanja B Russell J Adoption, non-adoption, and abandonment of a personal electronic health record: case study of HealthSpace BMJ 2010 341 c5814 10.1136/bmj.c5814 21081595 PMC2982892

[B13] Holden RJ Karsh BT The technology acceptance model: its past and its future in health care J Biomed Inform 2010 43 1 159 172 10.1016/j.jbi.2009.07.002 19615467 PMC2814963

[B14] Davis FD Bagozzi RP WP R User acceptance of computer technology: a comparison of two theoretical models Manag Sci 1989 35 8 982 1003 10.1287/mnsc.35.8.982

[B15] Venkatesh V Morris MG Davis FD User acceptance of information technology: Toward a unified view Management of Information System Research Center 2003 27 3 451 481

[B16] Murphy CA Coover D Owen SV Development and Validation of the Computer Self-Efficacy Scale Educ Psychol Meas 1989 49 4 893 899 10.1177/001316448904900412

[B17] Igbaria M Iivari J The effects of self-efficacy on computer usage Omega 1995 23 6 587 605 10.1016/0305-0483(95)00035-6

[B18] Fishbein M Ajzen I Belief Attitude, Intention and Behavior: An Introduction to Theory and Research 1975 Reading, MA: Addison-Wesley

[B19] Corey M Angst, Ritu Agarwal Adoption of electronic health records in the presence of privacy concerns: the elaboration likelihood model and individual persuasion MIS Q 2009 33 2 339 370

[B20] Ing-Long W Jian-Liang C An extension of Trust and TAM model with TPB in the initial adoption of on-line tax: an empirical study Int J Human-Computer Studies 2005 62 784 808 10.1016/j.ijhcs.2005.03.003

[B21] Cheng TCE Lam DYC Yeung ACL Adoption of internet banking: an empirical study in Hong Kong Decis Support Syst 2006 42 3 1558 1572 10.1016/j.dss.2006.01.002

[B22] David C Kaelber MD Ashish K Douglas J Blackford M Dacid WB A research agenda for personal health records (PHRs) J Am Med Inform Assoc 2008 15 6 729 736 10.1197/jamia.M2547 18756002 PMC2585530

[B23] Rigopoulos G A tam framework to evaluate users' perception towards online electronic payments Journal of Internet Banking & Commerce 2007 12 3 1 6

[B24] Ma WW Andersson R Streith KO Examining user acceptance of computer technology : an empirical study of student teachers J Comput Assist Learn 2005 21 6 387 395 10.1111/j.1365-2729.2005.00145.x

[B25] Wöber K Gretzel U User acceptance of information technology: Toward a unified view J Travel Res 2000 39 2 172 181 10.1177/004728750003900207

[B26] Maloney FL Wright A USB-based Personal Health Records: an analysis of features and functionality Int J Med Inform 2010 79 2 97 111 10.1016/j.ijmedinf.2009.11.005 20053582

[B27] Ajzen I Kuhl J, Beckman J From Intentions to Actions: A Theory of Planned Behavior 1985 Heidelberg: Springer 11 39

[B28] Kahn JS Aulakh V Bosworth A What it takes: Characteristics of the Ideal PHR Health Aff 2009 28 2 369 376 10.1377/hlthaff.28.2.369 19275992

[B29] Hubona GS Geitz S External Variables, Beliefs, Attitudes and Information Technology Usage Behavior30th Hawaii International Conference on System Sciences (HICSS) 1997 3 21 28

[B30] Rosenzweig MR Why are there returns to Schooling? Am Econ Rev 1995 85 2 153 158

[B31] Agarwal R Prasad J Are individual differences germane to the acceptance of new information technologies? Decis Sci 1999 30 2 361 391 10.1111/j.1540-5915.1999.tb01614.x

[B32] Adamson I Shine J Extending the new technology acceptance model to measure the end user information systems satisfaction in a mandatory environment: a bank's treasury Technology Analysis & Strategic Management 2003 15 4 441 455 10.1080/095373203000136033

[B33] Venkatesh V Davis FD A model of the antecedents of perceived ease of use: Development and Test Decis Sci 1996 27 3 451 481 10.1111/j.1540-5915.1996.tb01822.x

