# Supplementary material for: Factors influencing consumer adoption of USB-based Personal Health Records in Taiwan
Source: BMC Health Serv Res. 2012 Aug 27;12:277. doi: 10.1186/1472-6963-12-277 (PMC3465237; doi:10.1186/1472-6963-12-277)
Supplement: Additional file 1 — Appendix. [file 1472-6963-12-277-S1.doc]

**Appendix**

**Questionnaire**

The USB-PHR system is a portable personal health summary that stores a minimal data set of medical information essential for providing health care. The USB flash drive given to the patients contains a software to collect and view their personal medical information such as medication, lab results, multimedia files like endoscopy or ultrasound scanning recordings from hospitals they received treatment. Information will be updated from reception desk when the patients are discharged or after outpatient visit from the participating hospitals. USB-PHR contains two components, namely, XML data component and the viewer. The data component is a set of XML files compliant to a standard called TMT (Taiwan electronic Medical records Template) which is a derivative of HL7 CDA-R2. This XML data component helps in collecting relevant information from hospitals’ information system. Using the USB-PHR the patient can view the encrypted information that is provided by participating hospitals. Figure 1 shows the information collection from difference hospitals.


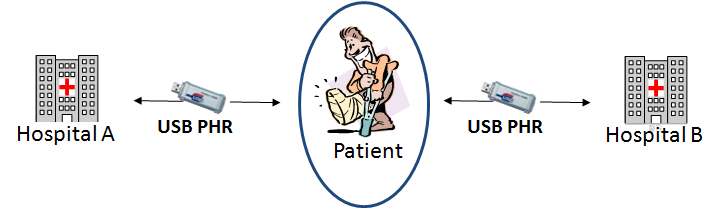


Figure 1 showing information collection from different hospitals

Figure 3 is screenshot of the viewer showing personal lab result when User plug-in USB in to PC or laptop. A new folder will be created by system for each hospital. Information from different departments will be stored in sub-folders pertaining to that hospital according to dates the patient visited as shown in figure 2. Patient or care provider has to just plug in and click on interested folder in order to view your medical information.


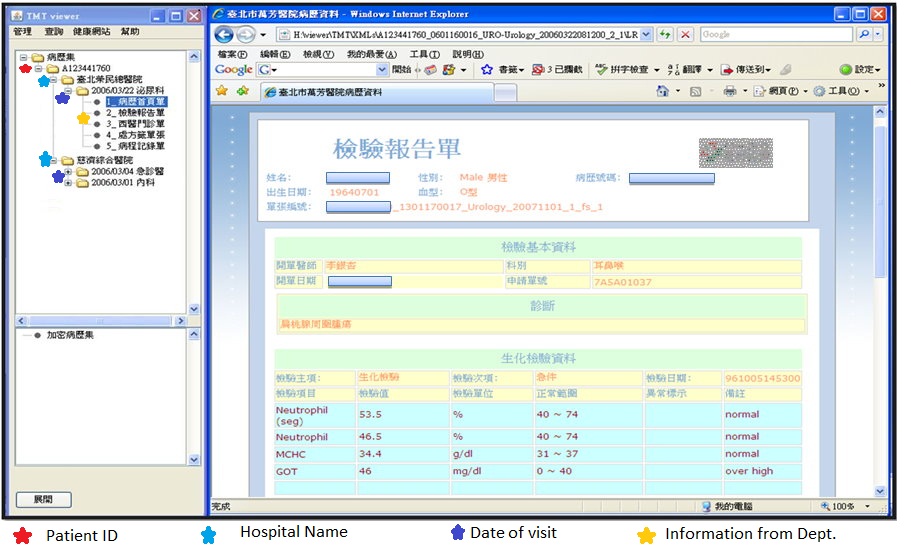


Figure 2. User Interface of PHR displaying lab results.

| **Part I. Cognition of USB Personal Health Record**  **(Perceived usefulness, Easy of use and Subjective norm)**  According to your understanding of USB Personal Health Record (USB-PHR). Please give a check mark “🗸” on the blank appropriately. | Strongly Agree | Agree | Neutral | **Disa**gree | Strongly Disagree |
| --- | --- | --- | --- | --- | --- |
| 1. Compared with paper-based, USB-PHR always facilitates obtaining my past medical history anytime anywhere. | □ | □ | □ | □ | □ |
| 1. USB-PHR allows me to better understand my diagnosis, plan of treatments and medication schedules. | □ | □ | □ | □ | □ |
| 1. USB-PHR helps me when I visit a new physician to comprehend my health condition. | □ | □ | □ | □ | □ |
| 1. My physician might reduce duplication of examinations and lab tests if I am using a USB-PHR. | □ | □ | □ | □ | □ |
| 1. It is not difficult for me to read information from the USB. | □ | □ | □ | □ | □ |
| 1. It is not difficult for me to carry a USB-PHR when I do a hospital visit. | □ | □ | □ | □ | □ |
| 1. I will be using an USB-PHR when my relatives, friends or colleague are using it. | □ | □ | □ | □ | □ |
| 1. I will be using an USB-PHR when my physician recommends and when government and hospital promote it. | □ | □ | □ | □ | □ |

| **Part II. Privacy and Security**  According to your notion of privacy and safety with the USB-PHR, please give a check mark, “🗸”on the blank appropriately. | Strongly Agree | Agree | General | Disagree | Strongly Disagree |
| --- | --- | --- | --- | --- | --- |
| 1. I believe USB-PHR can ensure data security by **digital signature** and timestamp. | □ | □ | □ | □ | □ |
| 1. I believe USB-PHR can ensure my personal security, if it is password protected | □ | □ | □ | □ | □ |
| 1. I believe Hospital policies will ensure my information stored in USB-PHR, only to be used in medical services. | □ | □ | □ | □ | □ |
| 1. I am not worried about privacy issues when using USB-PHR | □ | □ | □ | □ | □ |

| **Part III. Intention of Use**  According to your intention of using the USB-PHR, please give a check mark, “🗸”on the blank appropriately. |
| --- |
| 1. Do you intent to use USB-PHR? □ Yes　　 □ No 2. Do you apply for the USB-PHR now? 　□ Yes　　 □ No |

| **Part IV. Self-** **capability of using computer**  According to your confidence of self- capability of using computer, please give a check mark, “🗸”on the blank appropriately. | Strongly Agree | Agree | General | Disagree | Strongly Disagree |
| --- | --- | --- | --- | --- | --- |
| 1. I have the ability to retrieve information I need using the Internet | □ | □ | □ | □ | □ |
| 1. I have the ability to use computer software, such as MS Word, Excel and to save and retrieving files I need from a Personal Computer | □ | □ | □ | □ | □ |
| 1. I routinely use computer at my work. | □ | □ | □ | □ | □ |

| **Part V. Personal Information** |
| --- |
| 1. Gender: □ Male　□ Female 2. Year of Birth: 3. Education: □(1) Junior High School and below   □(2) High School/ Trade School □(3) Two-year College  □(4) Bachelor □(5) Master and above 4. Occupation: □(1) Worker □(2) farmer/ Fisherman/ Herdsman   □(3) Businessman □(4) Military/ Civil service □(5) Retirement   □(6) Housekeeper □(7) Services □(8) Student   □(9) Other＿＿＿ 5. Have you ever applied paper-based medical record before?　□Yes　　□ No 6. Have you ever been hospitalized or referral?　□(1)Yes　　□(2)No 7. Have you ever visited two or more hospitals within 6 months?　□(1)Yes □(2)No 8. Generally, how do you think about your health condition now?   　　□(1) Excellent　　□(2) Very Good　　□(3) Good　　□(4) Fine　　□(5) Bad |
